# Clinical Isolate of *Candida tropicalis* from a Patient in North Carolina: Identification, Whole-Genome Sequence Analysis, and Anticandidal Activity of *Ganoderma lucidum*

**DOI:** 10.4236/ojmm.2025.151002

**Published:** 2025-03-05

**Authors:** Akamu Ewunkem, Lydia Merrills, Zahirah Williams, Lisa Maness, Jeffery Meixner, Brittany Justice, Uchenna Iloghalu, Vera Williams, Larisa Kiki, Dinesh Singh

**Affiliations:** 1 Department of Biological Sciences, Winston Salem State University, Winston-Salem, USA; 2 Department of Nursing, Winston Salem State University, Winston-Salem, USA; 3 Department of Medical Laboratory Sciences, Winston Salem State University, Winston Salem, USA; 4 UNC Health Care Hillsborough, Hillsborough, USA; 5 North Carolina A and T State University, Greensboro, USA

**Keywords:** *Candida* sp., *Candida tropicalis*, Mushroom Extracts, Germ Tube, Resistance, Clinical Isolates

## Abstract

In North Carolina, candida infections are on the rise and pose a significant threat to human health in clinical settings. In addition, the rise of resistance to antifungal drugs has only heightened this concern. Importantly, misidentification of *Candida* spp. may result in underdiagnosis, patients getting the wrong treatment and incomplete infection prevention measures. The correct and rapid etiological identification of *Candida* infections is of paramount importance because it provides adequate therapy, reduces mortality, and controls outbreaks. Hence, this study aimed to identify *Candida* sp. up to species level of a clinical isolate from an infected patient treated in North Carolina using biochemical and molecular techniques. Due to the emergence of resistance, we explored whole genomic analysis to highlight polymorphisms that can impact candida resistance. Exploration for the effectiveness of bioactive compounds in natural products to treat *Candida* spp. resistant to present-day drugs could provide promising new treatment options for managing infected patients. Thus, this study also investigated anticandida activity of three solvent extracts of *Ganoderma lucidum* against the clinical isolate of *Candida* sp. The findings of this study provided evidence that *Candida tropicalis* MYA-3404 was the only strain present in the clinical isolate. The whole genome sequencing of *C. tropicalis* identified mutations in genes that most likely underscore drug resistance. All extracts from *G. lucidum* significantly (P < 0.05) inhibited the growth of *C. tropicalis*. Together, this work highlights the enormous potential of biochemical and molecular techniques in identifying clinical isolates of candida to species level and the use of bioactive compounds from extracts of *G. lucidum* as promising anticandidal agents. Further testing is needed to confirm the phenotypic expression of resistance.

## 1. Introduction

*Candida* infection is a serious opportunistic infection, which affects the oral cavity, vaginal, skin, stomach, and other parts of the body [1]. Untreated candida infection may affect vital organs like the heart, brain and kidneys, causing life-threatening complications [2]. The rate of candida infection is increasing rapidly and close to 1.5 million people have invasive candidiasis each year, with 995,000 deaths (63.6%) [3]. Severe candidiasis can be life threatening to patients with a weak immune system due to the body’s reduced ability to fight infections. This lucidly explains why 90% of patients with HIV/AIDS develop candidiasis [4]. Candidiasis because of HIV remains a persistent problem in the US.

About 25,000 cases of candidiasis occur in the US annually with a 25% mortality rate during hospitalization [5]. The Centers for Disease Control and Prevention (CDC) recently issued a warning about the increase in cases across the US. This is a cause of concern because patients in the health care setting are more susceptible. *Candida* infections have been making headlines in North Carolina since February 2023. According to the North Carolina Department of Health and Human Services, new cases of a multi-drug-resistant *Candida* were reported in North Carolina in February 2023. All reported cases occurred in patients with serious comorbid health conditions and/or a history of prolonged hospitalization. *Candida* infections can spread easily in healthcare settings causing invasive infections associated with mortality rates of up to 60% [6].

Isolation and identification of *Candida* spp. are very important because they can initiate prompt appropriate treatment fundamental to proper management of infected patients [7]. Identification of Candida at the species level is necessary to help in selecting the appropriate medication for the treatment. Traditionally, germ tube test and chromogenic culture media, have been used to identify *Candida* spp since they are rapid, easy-to-perform methods and allow for quick preliminary identifications of potential *Candida species* in clinical samples. However, these methods only reliably identifying *Candida albicans* and not other *Candida* species, such as *C. tropicalis*. Further confirmatory tests, like biochemical or molecular methods, are needed for accurate identification of *C. tropicalis*. Misidentifying *Candida species* can lead to a patient getting incorrect medication or treatment potentially causing serious complications for the patient due to ineffective or suboptimal antifungal therapy.

Recommended antifungal treatment for candida infections are synthetic antifungal drugs such as nystatin, clotrimazole, amphotericin B, and miconazole [8]. However, there is a steady increment in resistance to these antifungal medications. Resistance is caused by the formation of biofilms in *Candida*, transition from planktonic to sessile and expression of resistance genes [9]. Antifungal resistance may account for the high rates of therapeutic failures and mortality frequently documented with those infections worldwide, which necessitates the search for natural products capable of combating candida infections.

Mushrooms have been shown to be some of the best sources of natural antimicrobials. Consumption of mushrooms has increased because of consumer preference of microbiologically safe food [10]. Mushrooms have also piqued the interest of scientists and the clinical community for two major reasons. Mushrooms can modulate the immune system and are efficacious against numerous diseases due to the presence of a plethora of bioactive compounds known to inhibit mycelial growth of pathogens [11]-[14]. *Ganoderma lucidum*, commonly known as the reishi, varnished conk, or ling chih, is currently one of the most widely used mushrooms [15] [16]. *G. lucidum* is used as nutraceuticals, functional foods and as alternatives to antioxidants and antimicrobial agents [14] [17]-[19]. An analysis of *G. lucidum* extract revealed a range of biologically active compounds (such as polysaccharides, triterpenes, flavonoids, alkaloids, steroids ganoderic acid and less beta [1]-[3] glucan) with medicinal properties which have been the focus of many studies [14] [19]. These compounds are shown to display anti-inflammatory and anticancer properties, antifibrotic effects, cytotoxic and inhibitory activities [20] [21]. Early studies in our laboratory showed that alcohol, aqueous and dual solvent extracts of *G. lucidum* inhibited to different extents Gram-positive and Gram-negative bacteria; the ethanolic extract exhibited the most potent antimicrobial activity [19]. In other previous studies, the aqueous extract of *G. lucidum* showed the highest antimicrobial activity of *Candida albicans* [22] [23]. There are no previous studies indicating that *G. lucidum* has an ability to kill or inhibit the growth of candida that are directly collected from infected patients (clinical isolates) in a laboratory setting. In contrast other mushroom species, for example *Trametes* spp. and *Microporus* spp. have demonstrated antimicrobial activity against clinical isolate of *Candida albicans* [24]. To the best of our knowledge, no studies have reported on the antimicrobial activity of alcohol, aqueous and dual solvent extracts of *G. lucidum* extracts against clinical isolation of *Candida tropicalis* from a hospital patient. In this study, the antimicrobial activity of alcoholic, aqueous and dual solvent extracts from wild *G. lucidum* was investigated against clinical isolate of *Candida* sp. from a hospital patient in North Carolina, United States of America. The study highlighted the phenotypical and molecular traits for identifying *Candida* spp. isolates. In addition, whole genome sequencing was used to identify mutations in resistance genes in the clinical isolate of *Candida tropicalis*. Identifying mutations in resistance genes allows scientists and health personnels to understand the mechanisms of drug resistance, predict the emergence of resistant strains, guide treatment decisions by selecting the most effective antifungal agents against specific resistance profiles, and develop new therapeutic strategies to combat the growing problem of antimicrobial resistance.

## 2. Materials and Methods

### 2.1. Collection, Identification of *Ganoderma lucidum*

The wild mushrooms of *G. lucidum* were collected from their natural habitats in Winston Salem, North Carolina, USA in August 2023. The demonstration samples were preserved in the Antimicrobial and Genomics Lab in the Department of Biological Sciences, Winston Salem State University where standard keys were used to identify the mushrooms.

### 2.2. Preparation of *G. lucidum* Mushroom Extracts

Fresh *G. lucidum* mushrooms were harvested, chopped, and dried in an oven at 40°C - 45°C. The dried fruit body was blended to obtain mushroom powder. Mushroom extraction was carried out using 80% ethanol (Fisher Scientific, USA) and distilled water solvents following the protocol of Ewunkem *et al.* [19]. Briefly, the mushroom powder was weighed (100 g) and extracted by stirring with 500 ml of 80% ethanol or distilled water at 50°C for 48 hours. Dual extraction was prepared by initial extraction in ethanol (Fisher Scientific, USA) at 50°C for 48 hours and finally in equal volume of distilled water at 50°C temperature for 48 hours. The obtained extracts were separated through centrifugation (4200× g, 30 min) and stored at 4°C in 1L amber bottles prior to analysis.

### 2.3. High Performance Liquid Chromatography (HPLC) Analysis

HPLC analysis determined the bioactive compounds of aqueous and ethanol extracts of *G. lucidum* as described previously [14] [19]. HPLC was carried out on an Agilent 1100 series system equipped with a quaternary rapid separation pump (LPG-3400RS) and photodiode array detector (DAD-3000RS). Chromatography was performed in isocratic mode and the mobile phase consisted of HPLC water (A) and acetonitrile (B) using a gradient program at a flow rate of 1 mL/min. Aqueous extract and alcohol extracts of *G. lucidum* contents were determined based on the standard curves. Separation was performed using C18 column (4.6 mm × 250 mm i.d., 5 μm) at 35°C with a flow rate of 1 ml/min. The multi-wavelength detector was monitored at 280 nm.

### 2.4. Tested Microbial Strains and Culture Conditions

The *Candida* sp. used in this study was isolated from a patient with suspected candida infection treated at Wake Forest Medical Center, Winston Salem State University and stored at −80°C in tryptic soy broth with 20% glycerol for further investigation. The clinical isolate was a gift from the Department of Medical Laboratory Science (MLS), Winston Salem State University. To safeguard patients’ confidentiality, the department policy strictly prohibits the collection of any patient identifiers, including names, medical record numbers, dates of birth, contact information, or any other details that could potentially lead to patient identification to ensure that Health Insurance Portability and Accountability Act (HIPAA) is not violated. HIPAA is a federal law that protects the security and confidentiality of a patient’s health information. The candida strain was cultured on Potato Dextrose Agar (PDA), that consisted of the following ingredients in (g/L): Potato extract (200), dextrose (20) and agar (15) at a temperature of 35°C and preserved in Potato Dextrose Broth (PDB) supplemented with 50% glycerol at a temperature of −80°C for subsequent analysis.

### 2.5. Isolation and Identification of the *Candida* sp. Clinical Isolates

The *Candida* strain was directly inoculated onto blood agar (Fisher Scientific, USA) using 10 μL the culture YEPD broth and incubated at 35°C for 24 hours. Additional identification was performed utilizing the germ test where a suspension was made in a tube containing 0.5 mL of human plasma with a colony of *Candida* sp. sample and incubated at 35°C for 3 h after which a drop of suspension was placed on labeled microscope slides for examination of blastoconidia. To examine cell morphology, samples were prepared as described previously, Ewunkem *et al.* [19]. Briefly, morphotypes of the strain were fixated with 2.5% glutaraldehyde in 0.1 M phosphate buffer (pH 7.2) for overnight at 4°C followed by series of ethanol gradients: 0%, 50%, 70%, 80%, 90%, and twice with 100%. Then, the samples were dehydrated with acetone twice for 30 seconds until dried by the critical point method in liquid CO_2_. Subsequently, the specimens were coated with gold in a vacuum evaporator and examined with a scanning electron microscope (JEOL’s new Field Emission Scanning Electron Microscope, the JSM-IT800). The identification of *Candida* sp. was further confirmed by Multilocus sequence typing (MLST), a highly accurate and portable system for distinguishing between isolates of a microbial species as previously described [25].

### 2.6. Molecular Characterization of Clinical Isolates of *Candida* sp.

Candida isolation and purification of genomic DNA were performed at SeqCoast Genomics, Portsmouth, New Hampshire, USA. SeqCoast Genomics is an expert-guided genomics center offering microbial and small genome sequencing and bioinformatic analysis services. Isolation and purification were done using the DNeasy 96 PowerSoil Pro QIAcube HT Kit (Qiagen 47021, Hilden, Germany) as per manufacturer instructions. Strain identification based on sequencing was performed on the Illumina NextSeq2000 platform using a 300-cycle flow cell kit to produce 2x150bp paired reads. A quality check for raw reads was performed with FASTQC and reads were trimmed and paired using Trimmomatic (version 0.39.0) and Breseq (version: 0.37.0) [26].

### 2.7. Determination of Antimicrobial Activities of *G. lucidum*

Antimicrobial effects of ethanol extract, aqueous and dual extracts of *G. lucidum* mushroom against *Candida* sp. were investigated using broth microdilution method in 96-well plates. 200 ul of nutrient broth containing appropriate concentration of the extract of *G. lucidum* was added to each well, except for the controls. Next, a 10 μL suspension of microbial cells (8 × 10^6^ cells/mL) was added to each well; the sterility control well contained 100 mL of the nutrient broth, and the growth control well had 190 μL of medium and 10 μL of the candida suspension. The test was carried out in triplicate. The 96-well plates that contained *Candida* sp. were incubated at 35°C. The growth was assessed by measuring turbidity at 600 nm for hours 0, 3 and 24 h using a Glomax multi plate reader (Promega, USA). Examination of the morphological changes of *Candida* sp. was performed using a scanning electron microscope (SEM) following the methods described by Ewunkem *et al.* [14] and Ewunkem *et al.* [19].

## 3. Results

### 3.1. HPLC of Aqueous and Ethanol Extracts of *G. lucidum*

HPLC analysis of aqueous and ethanol extracts of *G. lucidum* extracts showed the presence of various bioactive compounds [19]. The three predominant bioactive compounds included beta [1]-[3] glucans, ganoderic acid and triterpenoids, with beta [1]-[3] glucan being most abundant followed by ganoderic acid.

### 3.2. Identification of *Candida* sp.

**Figure 1.**
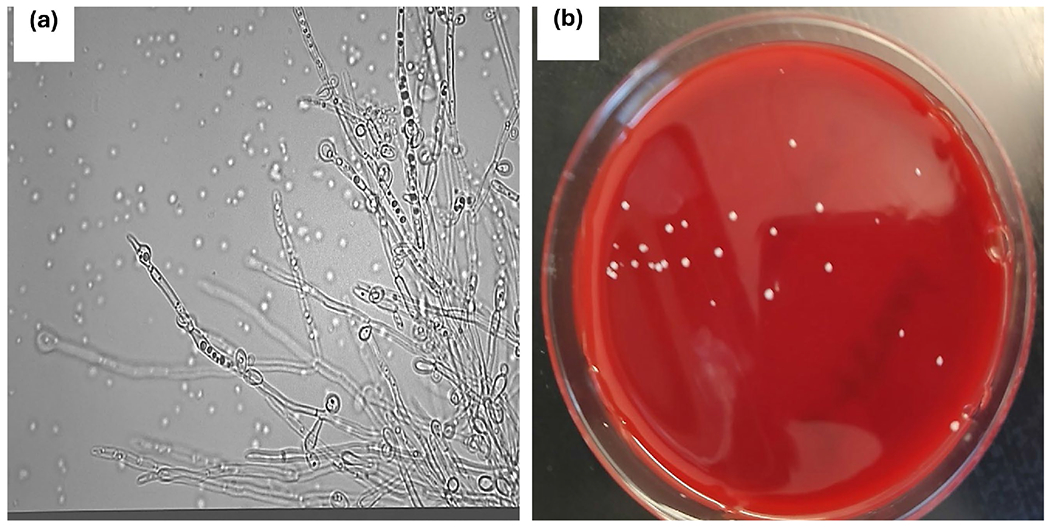
The morphological features of *Candida tropicalis* recovered from clinical specimens at the Wake Forest Medical Center, Winston Salem, North Carolina USA (a) *Candida tropicalis* showing blastoconidia in single or branched chains, with abundant pseudohyphae in human plasma under a light microscope (400×); (b) Cream-colored smooth colonies, after 24 h of incubation at 35°C grown on the blood agar.

**Figure 2.**
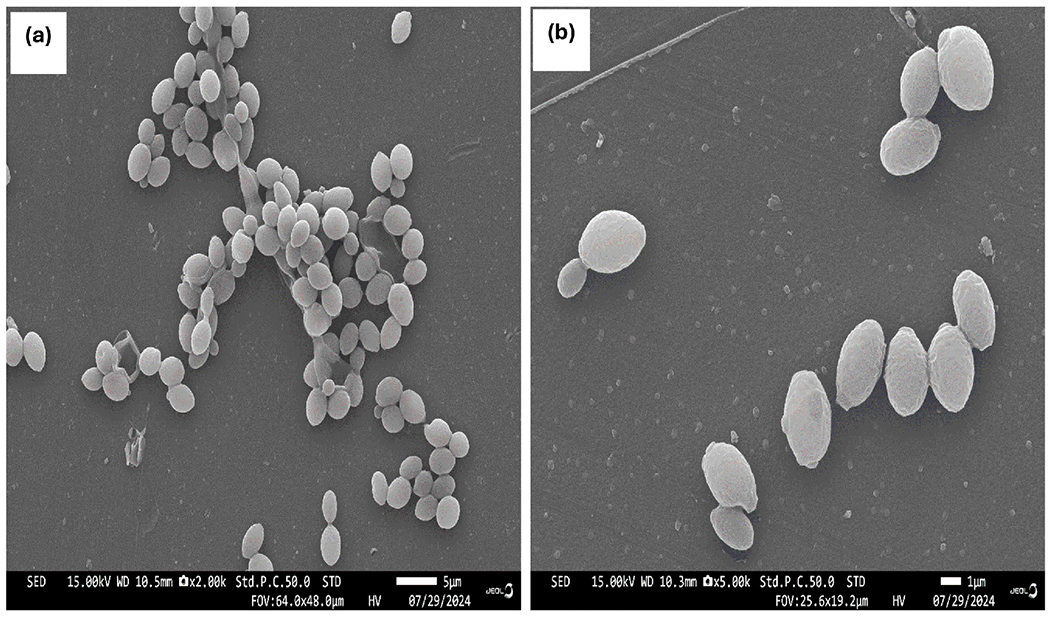
Scanning electron micrographs of *Candida tropicalis* clinical strain’s morphologies at ×2.00 k (A) and at ×5.00 k (B). *C. tropicalis* shows budding phenotype after 24 h incubation at 35°C in YEPD medium.

Identification of *Candida* sp. was carried out using phenotypic and molecular methods. When grown in human plasma at 37°C for 3 hours, they did not form germ tubes, detected as hyphae with no constrictions where the structures join the blastoconidia (Figure 1(a)). The blastoconidia were typically about around 2-5 micrometers in diameter, appeared as budding oval yeast-like cells under microscopy suggesting *Candida tropicalis*. When grown on bacteriological media (sheep blood agar and Nutrient broth agar), a presumptive identification of *Candida tropicalis* was made when they appeared as small, round, creamy colonies on blood agar (Figure 1(b)). SEM analysis of the clinical isolates of *Candida* sp. revealed round to oval shaped smooth-walled bodies less than 5 μM (Figure 2). The molecular results confirmed that *C. tropicalis* was the only strain found in the clinical sample (Table 1).

### 3.3. Whole-Genome Sequencing

To investigate the genetic characteristics of *C. tropicalis*, the genome of *C. tropicalis* was sequenced and analyzed to detect any polymorphisms associated with multidrug resistance. Polymorphisms were observed with all frequencies of mutations and locations reported in Table 2. Specifically, these polymorphisms occurred in: staphyloferrin A export MFS transporter/D-ornithine--citrate ligase SfaD (*sfaA/sfaD*); adenine phosphoribosyltransferase (*KQ76_RS08360*); teichoic acid D-Ala esterase FmtA (*fmtA*); DUF3169 family protein (*KQ76_RS01520*); alpha/beta hydrolase (*KQ76_RS13020*); DNA-binding heme response regulator HssR (*hssR*); ribosome biogenesis GTPase YlqF (*ylqF*); PG: teichoic acid D-alanyltransferase DltB (*dltB*); staphyloferrin A export MFS transporter/D-ornithine-citrate ligase SfaD (*sfaA/sfaD*); M23 family metallopeptidase/HAD-IIB family hydrolase (*KQ76_RS11280/KQ76_RS11285*); general stress protein (*KQ76_RS01815*); SsrA-binding protein SmpB (*smpB*); phage major capsid protein (*KQ76_ RS07375*); hosphoribosylformylglycinamidine synthase subunit PurS (*purS*); tRNA uridine-5-carboxymethylaminomethyl(34) synthesis enzyme MnmG (*mnmG*); alpha/beta hydrolase (*KQ76_RS13020*); D-lactatedehydrogenase (*KQ76_RS12955*); BglG family transcription antiterminator (*KQ76_RS10985*); DNA-binding heme response regulator HssR (hssR); magnesium transporter CorA family protein (*KQ76_RS12180*) and magnesium transporter CorA family protein (*KQ76_RS12180*).

### 3.4. Anticandidal Activity of Aqueous Ethanol and Dual Extracts of *G. lucidum* Against *C. tropicalis*

**Figure 3.**
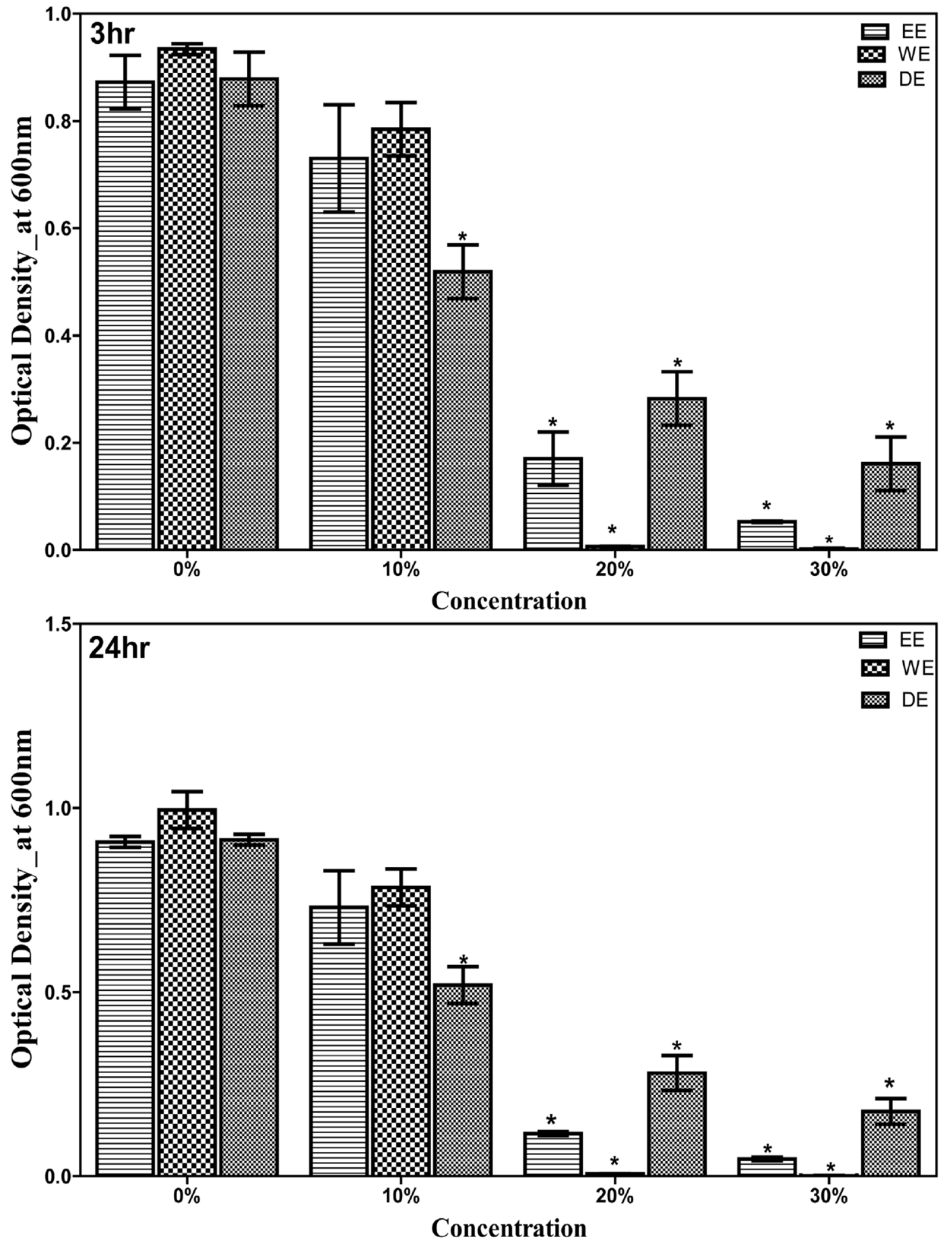
Antimicrobial activity of ethanolic, aqueous, and dual solvent extractions of *G. lucidum* against *C. tropicalis* after 3 h and 24 h of incubation. The growth of *C. tropicalis* was measured by a spectrophotometer at 600 nm wavelength. EE = ethanol extract; WE = water extract or aqueous extract; DE = dual extract. Asterix (*) indicates significant difference compared with control (P < 0.05).

The effectiveness of aqueous, ethanol and dual extracts of *G. lucidum* against *C. tropicalis* was observed within the first 3 hours of incubation. The anticandidal activity of each extract of *G. lucidum* increased with concentration (0% < 10% < 20% - 30%) (Figure 3). Each tested concentration exhibited significant (P < 0.05) effect over the control. Overall, the most effective concentration on the *C. tropicalis* was observed with the aqueous extract.

### 3.5. Scanning Electron Microscopy (SEM) Observation

**Figure 4.**
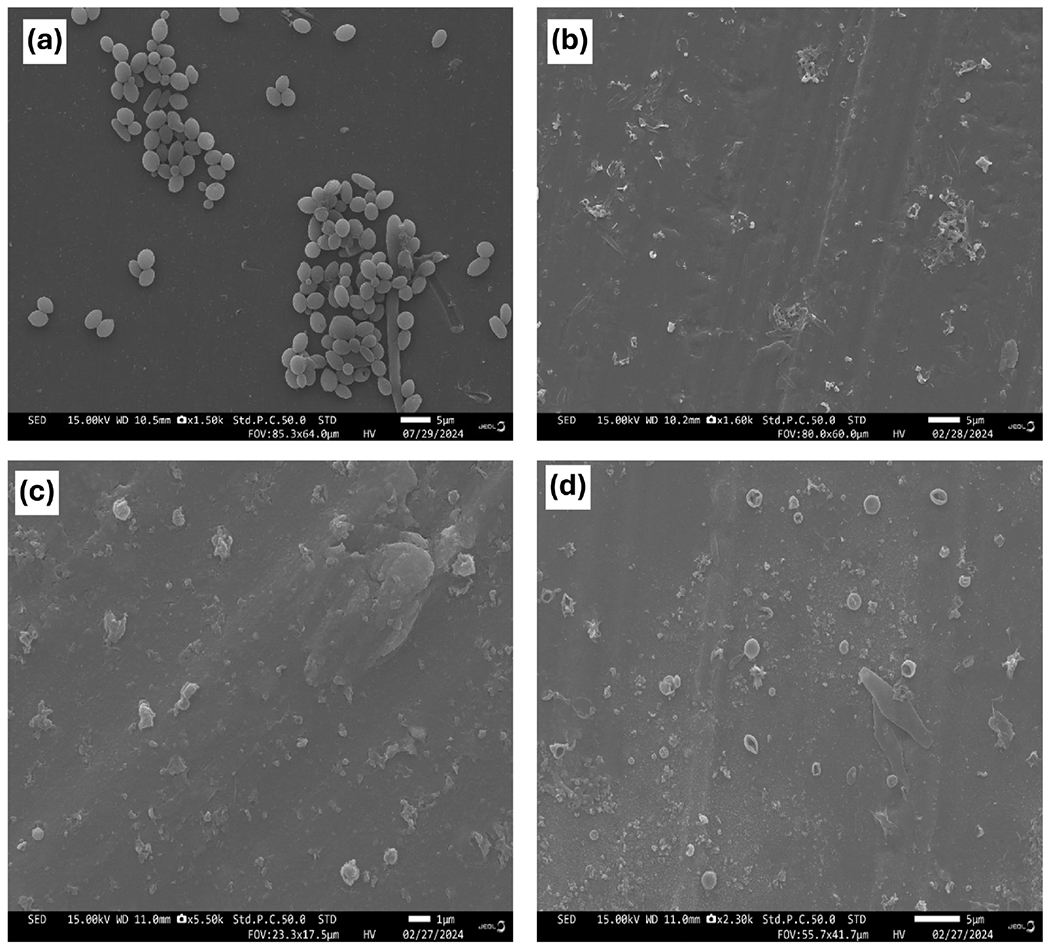
Scanning electron microscopy micrographs of *C. tropicalis* and control groups after incubation with various *G. lucidum* extracts at 24 h. (a) *C. tropicalis* control cell with normal surface; (b) ethanol extract treated *C. tropicalis* with surface irregularities; (c) water extract treated *C. tropicalis* with surface irregularities; (d) dual solvent extraction treated *C. tropicalis* with surface irregularities.

Given the antimicrobial efficacy of *G. lucidum* against *C. tropicalis*, the morphological features of *C. tropicalis* before and after exposure to the aqueous, ethanol and dual extracts *G. lucidum* were evaluated by SEM. The SEM images of treated *C. tropicalis* cells or untreated with *G. lucidum* are shown in Figure 4. In the untreated *C. tropicalis* cells, a smooth and intact surface was clearly visible (Figure 4(a)). On the other hand, considerable morphological changes including cell membrane damage, collapse in their cell wall, deformation and shrinkage were seen in *C. tropicalis* treated with *G. lucidum* (Figures 4(b)–(d)).

## 4. Discussion

The rise of antibiotic-resistant candida infections is an emerging issue in North Carolina that presents a serious threat among hospital patients. *Candida* spp. colonize patients (especially those with multiple predisposing factors) for months to years and are often multidrug-resistant let alone difficult to identify with standard laboratory methods [6] [27]. Misidentification may sometimes lead to inappropriate management and cause multiple outbreaks in healthcare settings. In the present study, a clinical isolates of *Candida* sp. were obtained from a hospitalized patient diagnosed with candidiasis in Winston Salem, North Carolina, USA. Generally, isolation and identification of *Candida* spp. in a diagnostic laboratory includes culture, germ-tube generation in plasma, and molecular techniques [28]. We used three different methods (two phenotypic and one molecular) for the identification of a *Candida* sp. isolated from the clinical sample. A presumptive identification of *Candida tropicalis* was made by observing round and cream-colored colonies on blood agar after 24 h of incubation.

Blood agar is a rich, complex medium used in medical diagnostics labs to grow and identify various bacterial pathogens as well as rapidly growing fungi including *Candida*. Blood agar media are generally prepared using tryptic soy agar with either 5% of sheep, rabbit, or horse blood. Blood agar plates can also identify microbes that produce hemolysins, enzymes that damage erythrocytes. *Candida* strains are known to exhibit hemolytic activity thus restoring the transferrin-inhibited growth of *Candida*. Typically, the colonies of *Candida* sp. on blood agar vary in appearance depending on the strain [29]. For example, *C. tropicalis* has creamy colonies that differ from other *Candida* spp. *C. albicans* are creamy grey with projections, or “feet”, that extend from the colony margins into the agar while *C. glabrata* produces relatively pink or dark purple, small colonies with pigments that may diffuse into the surrounding agar.

The presumptive *C. tropicalis* that demonstrated characteristic morphology was examined by germ-tube test, microscopy, and molecular technique to confirm its positive identity in the patient’s sample. The germ tube test is a screening test or a diagnostic tool that helps differentiate *Candida* spp. [30]. Blastoconidia of *C. albicans* are typically 2 - 8 μm in diameter and larger than blastoconidia (1 - 4 μm) of *C. glabrata*. Blastoconidia of *C. dubliniensis* are smooth-edged and viscid, made up entirely of blastoconidia. It should be noted that the blastoconidia are noticeably smaller than the blastoconidia of *C. albicans* and may appear round or oval confirming our results (Figure 1(a)) [31]. In general *Candida* spp. that form blastoconidia are commensal in nature but become opportunistic pathogens causing opportunistic infections when the blastoconidia convert into the hyphal form through morphogenesis [32]. In addition, the transition of blastoconidia to hyphae is linked to resistance against certain antifungal drug due to cell wall changes, biofilm architecture, efflux pump and metabolic plasticity [33] [34]. Whole genome sequencing, therefore, will undoubtedly be a useful tool to pinpoint specific genetic sequences within the genome of *C. tropicalis* genome that code for resistance.

The whole genome sequencing analysis of the clinical isolate of *Candida* sp. was used to identify genetic mutations associated with resistance to antifungal drug. The clinical isolate contains four resistance genes, staphyloferrin A export MFS transporter/D-ornithine--citrate ligase (*sfaA/sfaD*); adenine phosphoribosyltransferase (*KQ76_RS08360*), Ribosome biogenesis GTPase YlqF (*ylqF*) and BglG family transcription antiterminator (KQ76_RS10985) [35]–[40]. Staphyloferrin A export MFS transporter/D-ornithine--citrate ligase (*sfaA/sfaD*) plays an important role in multidrug resistance by transporting substances either down their concentration gradient or uphill using the energy stored in the electrochemical [36] [41]. Ribosome biogenesis GTPase YlqF (*ylqF*) facilitates structural changes and protein interaction ensuring protein synthesis which is essential for Candida’s growth and survival [39]. Targeting ribosome biogenesis GTPases could be a potential strategy for developing new drugs against *Candida* infections [37]. BglG family transcription antiterminator (*KQ76_RS10985*) controls gene expression, adaptation, and the ability to overcome antifungal therapy [35] [40]. Polymorphisms in staphyloferrin A export MFS transporter/D-ornithine--citrate ligase (*sfaA/sfaD*); adenine phosphoribosyltransferase (*KQ76_RS08360*), Ribosome biogenesis GTPase (*ylqF*) and BglG family transcription antiterminator (*KQ76_RS10985*) contribute to antifungal resistance by allowing Candida to evade the drug’s effects, often through mutations in genes associated with drug uptake, efflux pumps, or the target molecule itself, leading to decreased susceptibility to antifungal medications. Studying genetic polymorphisms in Candida allows for better understanding of resistance mechanisms and helps in monitoring the emergence of resistant strains. Furthermore, insight into the genetic basis of resistance can guide the development of new antifungal drugs that target different pathways or overcome known resistance mechanisms. The fact that polymorphisms were found in the clinical isolate strengthens the argument that pathogenic candida represents one of the most frequent causes of fungal infections, associated with high morbidity and mortality in the healthcare system [41]. Antibiotic-resistant candida can be difficult to treat hence the use of natural products can be extremely successful in the discovery of new medicine. In this context, extracts of *Ganoderma lucidum*, which remain unexplored in this area, might be a valuable resource in the search of new bioactive extracts/compounds which have already demonstrated antibacterial activity [14] [19].

A whole genomic analysis also showed mutations in eight genes dedicated to transport and metabolism, synthesis, invasion, oxidative stress, and energy production. These genes included adenine phosphoribosyltransferase (*KQ76_RS08360*), DUF3169 family protein (*KQ76_RS01520*); alpha/beta hydrolase (*KQ76_RS13020*); DNA-binding heme response regulator HssR (*hssR*); Hosphoribosylformylglycinamidine synthase subunit PurS (*purS*); tRNA uridine-5-carboxymethylaminomethyl (34) synthesis enzyme MnmG (*mnmG*); D-lactate dehydrogenase (*KQ76_RS12955*); magnesium transporter CorA family protein (*KQ76_RS12180*). Adenine phosphoribosyltransferase (*KQ76_RS08360*) catalyzes a salvage reaction resulting in the formation of AMP, that is energetically less costly than *de novo* synthesis [42]. DUF3169 family protein (*KQ76_RS01520*) is required for penetration of the host cells [43]. Alpha/beta hydrolase (*KQ76_RS13020*) plays a pivotal role in lipid metabolism [44]. DNA-binding heme response regulator HssR (*hssR*) is a transcriptional activator that plays an important role in the activation of genes required for respiration and oxidative stress and genes necessary for uptake and utilization of iron [45] [46]. Hosphoribosylformylglycinamidine synthase subunit PurS (*purS*) is vital to produce purine nucleotides building blocks for DNA and RNA [47]. tRNA uridine-5-carboxymethylaminomethyl (34) synthesis enzyme MnmG (*mnmG*) is responsible for proper tRNA function in translation [48]. D-lactate dehydrogenase (*KQ76_RS12955*) functions as an enzyme that converts D-lactate into pyruvate, essentially allowing the fungus to utilize D-lactate as a carbon source for energy production, particularly in environments where other sugars might be limited; this process is important for its survival and adaptation to different host conditions [49]. Magnesium transporter CorA family protein (*KQ76_RS12180*) controls the transport of divalent ions such as magnesium ions across the membrane [50]. Typically, *Candida* and other yeast must adapt to various host microenvironments during colonization of the host. Furthermore, genetic mutations of candida genes underline the rapid evolution of this aggregative phenotype [51]. Mutations in these genes could have enhanced some important pathogenicity factors of candida which allow colonization, adhesion, invasion, and damage to the host tissues. Additionally, polymorphisms of genes associated with transport and metabolism, synthesis, invasion, oxidative stress, and energy production in the clinical isolate of *Candida* sp. might have enabled the pathogen to grow, survive, and adapt to changing environmental conditions. Therefore, healthcare facilities should monitor candida infections in patients for such polymorphisms. In our study a clinical isolate of *Candida* sp. was identified as *C. tropicalis*. *C. tropicalis* is known to colonize the ears, wounds, respiratory tracts, urinary tracts, and skin of immunocompetent individuals, and can also cause serious bloodstream and/or invasive infections largely in immunocompromised patient [52] [53].

Finally, mutations in four genes not associated with fungi were found in this clinical isolation of *Candida tropicalis*. One mutated gene was teichoic acid D-Ala esterase FmtA (*fmtA*), which is not typically found in fungi because teichoic acids are a unique structural component of Gram-positive bacteria cell walls. D-Ala esterase reduces the positive charge on the cell surface, impacting resistance to cationic antimicrobial peptides and contributing to overall bacterial survival [54]. PG:teichoic acid D-alanyltransferase DltB (*dltB*) is linked to bacterial virulence contributing to resistance against host immune responses [55]. M23 family metallopeptidase/HAD-IIB family hydrolase (*KQ76_RS11280/KQ76_RS11285*) plays a crucial role in breaking down the bacterial cell wall peptidoglycan by cleaving specific bonds within its structure [56]. SsrA-binding protein SmpB (*smpB*) has a high affinity to protect bacteria from degradation and prevent secondary structure formation [57]. Phage major capsid protein (KQ76_RS07375) protects and encapsulates the viral genome, forming the structural shell of the virus particle, allowing for delivery of the genetic material to a host cell [58].

The presence of mutations in genes associated with bacteria in Candida, known as “bacteria genes in *Candida*”, is due to complex interactions between bacteria and *Candida species* where certain bacterial factors can influence the behavior and virulence of *Candida*. This interaction is very common in mixed microbial communities like the gut microbiome where bacteria and *Candida* coexist and can influence each other’s activity which may facilitate virulence and pathogenicity in both pathogens. For example, the interactions can either enhance or suppress candida virulence depending on the specific bacterial species and environmental conditions [59]. For instance, the interaction between *C. albicans* and *Viridans streptococci* is important for candida colonization affect polymicrobial biofilm virulence mechanisms, adhesion, invasion, and antimicrobial resistance [60] [61]. These findings suggest that patients with Candida infections may have higher rates of bacterial infections. Understanding trans-kingdom interactions may help identify novel strategies to treat infectious diseases. The presence of bacterial genes in the patience samples may not only represent the presence of bacterial infections, it may also represent sample bacterial contamination in the hospital setting, as hospitals have a high prevalence of bacteria on surfaces and in the air, which can easily contaminate clinical isolates of Candida cultures if proper aseptic techniques are not followed during sample collection and handling; this can potentially compromise the accuracy of the Candida identification and susceptibility testing. This may require careful interpretation of the report, ranging from sample contamination to urinary tract infections, including disseminated candidiasis. Scanning Electron Microscopy (SEM) can provide strong visual clues.

In this study, scanning electron microscopy (SEM) was used to study the morphology of the clinical isolate of *Candida* sp. SEM has been used to analyze the morphologies of clinical isolates of Candida strains [62] [63]. SEM images of clinical isolates from our patients appeared round to oval-shaped yeast cells with filamentous structures (hyphae). These data are in accordance with the results generated by Ma *et al.* [64] who described the morphology of *C. albicans* and *C. tropicalis* using SEM. In the control cultures, C. *tropicalis* and *C. albicans* were round or oval with smooth surface and 2 - 5 μm in diameter. Strain typing is a highly accurate tool for detecting Candida strains in clinical isolates such as *C. albicans*, *C. glabrata*, and *C. tropicalis* and *C. krusei* [65]–[68]. In this study stain typing confirmed the presence of *C. tropicalis* MYA-3404 in the clinical isolate from the patient.

*C. tropicalis* MYA-3404 is a diploid opportunistic pathogenic yeast, whose genome size is 14.5 Mb and contains 6258 genes encoding proteins and a guanine-cytosine content of 33.1% [69]. C. *tropicalis* causes both superficial and systemic infections in humans and has been increasingly isolated as the cause of invasive candida infections, ranking as the third most isolated species in patients with *Candida* infection [70] [71]. *C. tropicalis* has various virulence factors such as the morphological transition from yeast cell to hyphae, the production of extracellular hydrolytic enzymes (such as phospholipase, proteinase and hemolysin), biofilm formation and phenotypic switching [72] [73].

*Candida* infections kill more than one million people each year and are challenging to eliminate despite the existence of antifungal treatments. Infections with *C. tropicalis* are treated with several classes of antifungal drugs such as azoles, echinocandins, and amphotericin B [74]. These drugs work by inhibiting enzymes that produce the fungal cell membrane, causing the membrane to become unstable and leak, leading to cell death [75]. Furthermore, some anticandidal drugs can target the synthesis of chitin, a major component of the fungal cell wall [76]. However, resistant strains of *C. tropicalis* are commonly detected in patients, demanding an urgent need for new treatments. Mushroom extracts are a potential alternative because they contain bioactive compounds and secondary metabolites such as beta [1]–[3] glucans, ganoderic acid and triterpenoids that can kill or inhibit the growth of pathogens [19] [77] [78]. The bioactive compounds can act individually or in synergism to inhibit growth [79]. The present study involved *G. lucidum* extracts using water, ethanol and dual extracts (combined water and ethanol) and investigated activity against *C. tropicalis*. The water extract showed strongest anticandidal activity compared to the water and dual extracts. This may be due to the higher proportion of water-soluble bioactive compounds in *G. lucidum* mushroom [80]. A previous study indicated that *G. lucidum* and *Pleurotus ostreatus* ethanolic extracts showed a greater antimicrobial activity than water extracts [81].

Antimicrobial activity of extracts can affect the morphology of cells often frequently causing visible changes [82]. Scanning electron microscopy (SEM) is used to visualize the morphological changes in cells exposed to antimicrobial extracts [19] [83]. In this study, SEM was used to confirm the antimicrobial activity of extracts against *C. tropicalis*. Regardless of the extraction method SEM images of *C. tropicalis* treated with these extracts showed altered morphology and loss of cell integrity. This can be explained by the interaction of the bioactive compounds [19] and metabolites of *G. lucidum* extracts with *C. tropicalis* organelles and cellular process. Studies have shown that bioactive compounds and secondary metabolites can disrupt membrane permeability or redox balance and inhibit cell wall synthesis [84]. These results are in agreement with Pereira *et al.*, [85] who showed that aqueous and methanolic extracts of *Macrocybe titans* extracts caused morphological changes and slight damage to *C. albicans*.

However, this study has potential limitations, for example, the study is based on testing an isolate obtained from a single patient instead of different clinical samples. A single isolate may not represent the entire candida population within a patient, potentially leading to misinterpretation of resistance patterns. Also, the site of sample collection can influence the type of Candida isolated, potentially not reflecting the full range of pathogens involved in an infection. Furthermore, a key a limitation of using clinical isolates is that they only represent a snapshot of the species of Candida present in a designated patient at a predetermined time, potentially not capturing the full spectrum of resistant strains. Resistant strains are influenced by antibiotic usage, leading to skewed susceptibility patterns if not carefully interpreted. The process of isolating and testing clinical isolates can be time-consuming and perhaps not always accurately reflect the *in vivo* dynamics of infection. *In vivo* antimicrobial susceptibility testing was performed in controlled laboratory conditions, which may not fully reflect the complex environment of the human body. Interpreting the antimicrobial results without considering the clinical context, such as patient factors and infection site, can lead to inappropriate treatment choice. No invitro tests were performed for antifungal sensitivity. We are currently performing some invitro antifungal sensitivity test to compare the invitro susceptibility data with the molecular findings.

## 5. Conclusion

In North Carolina, candida infections are an emerging public health concern due to their potential for multi-drug resistance. Moreover, candida infections can rapidly spread in health care settings, leading to outbreaks with high mortality rates among vulnerable patients. There are multiple species of candida that can cause infections in hospitals and correct identification can directly lead to better outcomes, ensuring that the patient receives the appropriate medical care, preventing potential errors like administering wrong medications. Specific identification of candida is crucial for optimal treatment. This study aimed to identify *Candida* up to species level of a clinical isolate from a patient treated in a local medical center in Winston Salem, North Carolina. Additionally, whole genomic sequencing was performed to identify resistance genes. Moreover, the study assessed the antimicrobial activities of three solvent extracts of *G. lucidum* mushroom extract against the clinical isolate of *Candida* sp. The findings of this study provide evidence that *Candida tropicalis* MYA-3404 was the most prevalent *Candida species* in the clinical sample. The whole genome sequencing analysis of the *C. tropicalis* isolate revealed the occurrence of point mutations in genes involved in drug resistance. However, all the extracts of *G. lucidum* showed potent antimicrobial activities against *C. tropicalis* by altering morphology causing loss of cell integrity where antimicrobial efficacy is directly influenced by the bioactive compounds present in *G. lucidum*. Given that drug resistant candida infections are a growing health care problem, *G. lucidum* provides a new approach to treatment. Our study findings suggest several opportunities for additional study. Molecular studies and related techniques should be exploited to investigate the role of active components of *G. lucidum* extracts in controlling and preventing the proliferation of various cancer cells and diseases caused by viral pathogens. Further investigation is needed to perform antifungal sensitivity test and to evaluate the effects of extracts of *G. lucidum* on human cells.

## Figures and Tables

**Table 1.** Identification of Candida strains by strain typing.

# Assembly name: ASM633v3
# Organism name: *Candida tropicalis* MYA-3404 (budding yeasts)
# Infraspecific name: strain=MYA-3404
# Taxid: 294747
# BioSample: SAMN02953608
# BioProject: PRJNA13675
# Submitter: Broad Institute
# Date: 2009-06-18
# Assembly type: haploid
# Release type: major
# Assembly level: Scaffold
# Genome representation: full
# WGS project: AAFN02
# Genome coverage: 8.85x
# Sequencing technology: ABI
# RefSeq category: Representative Genome
# GenBank assembly accession: GCA_000006335.3
# RefSeq assembly accession: GCF_000006335.3
# RefSeq assembly and GenBank assemblies identical: yes
#
## Assembly-Units:
## GenBank Unit Accession
RefSeq Unit Accession
Assembly-Unit name
## GCA_000006345.3
GCF_000006345.3
Primary Assembly
## GCA_000348035.1
GCF_000348035.1
non-nuclear
#
# Ordered by chromosome/plasmid; the chromosomes/plasmids are followed by
# unlocalized scaffolds.
# Unplaced scaffolds are listed at the end.
# RefSeq is equal or derived from GenBank object.
#

# Sequence-Name	Sequence-Role	Assigned-Molecule	Assigned-Molecule-Location/Type	GenBank-Accn	Relationship	RefSeq-Accn	Assembly-Unit	Sequence-Length	UCSC-stylename
supercont3.1	unplaced-scaffold	na	na	GG692395.1	=	NW_003020038.1	Primary Assembly	2474448	na
supercont3.2	unplaced-scaffold	na	na	GG692396.1	=	NW_003020049.1	Primary Assembly	2308670	na
supercont3.3	unplaced-scaffold	na	na	GG692397.1	=	NW_003020055.1	Primary Assembly	2216334	na
supercont3.4	unplaced-scaffold	na	na	GG692398.1	=	NW_003020056.1	Primary Assembly	1654078	na
supercont3.5	unplaced-scaffold	na	na	GG692399.1	=	NW_003020057.1	Primary Assembly	1368217	na
supercont3.6	unplaced-scaffold	na	na	GG692400.1	=	NW_003020058.1	Primary Assembly	1255791	na
supercont3.7	unplaced-scaffold	na	na	GG692401.1	=	NW_003020059.1	Primary Assembly	920995	na
supercont3.8	unplaced-scaffold	na	na	GG692402.1	=	NW_003020060.1	Primary Assembly	802573	na
supercont3.9	unplaced-scaffold	na	na	GG692403.1	=	NW_003020061.1	Primary Assembly	498422	na
supercont3.10	unplaced-scaffold	na	na	GG692404.1	=	NW_003020039.1	Primary Assembly	430102	na
supercont3.11	unplaced-scaffold	na	na	GG692405.1	=	NW_003020040.1	Primary Assembly	419327	na
supercont3.12	unplaced-scaffold	na	na	GG692406.1	=	NW_003020041.1	Primary Assembly	125861	na
supercont3.13	unplaced-scaffold	na	na	GG692407.1	=	NW_003020042.1	Primary Assembly	22644	na
supercont3.14	unplaced-scaffold	na	na	GG692408.1	=	NW_003020043.1	Primary Assembly	12816	na
supercont3.15	unplaced-scaffold	na	na	GG692409.1	=	NW_003020044.1	Primary Assembly	11214	na
supercont3.16	unplaced-scaffold	na	na	GG692410.1	=	NW_003020045.1	Primary Assembly	11104	na
supercont3.17	unplaced-scaffold	na	na	GG692411.1	=	NW_003020046.1	Primary Assembly	8471	na
supercont3.18	unplaced-scaffold	na	na	GG692412.1	=	NW_003020047.1	Primary Assembly	8448	na
supercont3.19	unplaced-scaffold	na	na	GG692413.1	=	NW_003020048.1	Primary Assembly	7285	na
supercont3.20	unplaced-scaffold	na	na	GG692414.1	=	NW_003020050.1	Primary Assembly	6389	na
supercont3.21	unplaced-scaffold	na	na	GG692415.1	=	NW_003020051.1	Primary Assembly	6342	na
supercont3.22	unplaced-scaffold	na	na	GG692416.1	=	NW_003020052.1	Primary Assembly	5408	na
supercont3.23	unplaced-scaffold	na	na	GG692417.1	=	NW_003020053.1	Primary Assembly	4896	na
supercont3.24	unlocalized-scaffold	MT	Chromosome	GG692418.1	=	NW_003020054.1	non-nuclear	50304	na

**Table 2.** Genomics analysis of clinical isolate of *Candida tropicalis*.

Position	Freq (%)	Annotation	Gene	Gene product
2,228,365	69.2	intergenic (−35/−67)	*sfaA/sfaD*	staphyloferrin A export MFS transporter/D-ornithine--citrate ligase SfaD
1,700,478	65.6	G59S (GGC→AGC)	*KQ76_RS08360*	adenine phosphoribosyltransferase
1,017,325	65.3	Q304* (CAA→TAA)	*fmtA*	teichoic acid D-Ala esterase FmtA
342,330	62.0	I180I (ATT→ATC)	*KQ76_RS01520*	DUF3169 family protein
2,574,726	32.2	G69A (GGC→GCC)	*KQ76_RS13020*	alpha/beta hydrolase
2,389,192	32.0	R188P (CGA→CCA)	*hssR*	DNA-binding heme response regulator HssR
1,213,003	31.3	E184D (GAG→GAC)	*ylqF*	ribosome biogenesis GTPase YlqF
860,322	31.3	E344K (GAA→AAA)	*dltB*	PG:teichoic acid D-alanyltransferase DltB
2,228,366	30.8	intergenic (−36/−66)	*sfaA/sfaD*	staphyloferrin A export MFS transporter/D-ornithine--citrate ligase SfaD
2,252,747	29.9	intergenic (−54/+157)	*KQ76_RS11280/KQ76_RS11285*	M23 family metallopeptidase/HAD-IIB family hydrolase
391,361	29.1	S36I (AGT→ATT)	*KQ76_RS01815*	general stress protein
810,694	29.0	M1K (ATG→AAG)	*smpB*	SsrA-binding protein SmpB
1,536,460	27.5	Y112* (TAT→TAA)	*KQ76_RS07375*	phage major capsid protein
1,027,271	27.0	A87P (GCA→CCA)	*purS*	hosphoribosylformylglycinamidine synthase subunit PurS
2,776,116	26.7	H117Q (CAT→CAA)	*mnmG*	tRNA uridine-5-carboxymethylaminomethyl(34) synthesis enzyme MnmG
2,574,727	26.6	G69R (GGC→CGC) ‡	*KQ76_RS13020*	alpha/beta hydrolase
2,564,194	26.6	A17P (GCA→CCA)	*KQ76_RS12955*	D-lactate dehydrogenase
2,190,680	25.7	T500S (ACG→TCG)	*KQ76_RS10985*	BglG family transcription antiterminator
2,389,185	25.4	D186H (GAT→CAT)	*hssR*	DNA-binding heme response regulator HssR
2,408,432	25.4	E212Q (GAA→CAA)	*KQ76_RS12180*	magnesium transporter CorA family protein
